# AI-Powered Identification of Human Cell Surface Protein Interactors of the Hemagglutinin Glycoprotein of High-Pandemic-Risk H5N1 Influenza Virus

**DOI:** 10.3390/v17121638

**Published:** 2025-12-17

**Authors:** Christian Poitras, Benoit Coulombe

**Affiliations:** Montreal Clinical Research Institute, 110 Pine Ave West, Montréal, QC H2W 1R7, Canada; christian.poitras@ircm.qc.ca

**Keywords:** influenza A virus, H5N1, hemagglutinin, protein–protein interactions, artificial intelligence, AlphaFold, pandemic risk, antiviral targets

## Abstract

H5N1 is a highly pathogenic avian influenza virus of major global concern. Since 2023, it has circulated widely among wild and farmed birds, with increasing spillover into mammals, including minks, seals, and cattle, and sporadic infections in humans in Chile, the UK, and the USA. The risk of a future pandemic is considered high because ongoing viral evolution could enable efficient human-to-human transmission. The hemagglutinin (HA) glycoprotein is the principal determinant of host range, mediating viral attachment and entry through interactions with sialylated glycans and potentially additional host surface proteins. Here, we developed an artificial intelligence (AI)-based pipeline integrating structural modeling, protein–protein interaction prediction, and biological filtering to identify human cell surface proteins with high likelihood of interacting with H5N1 HA. These interactions may contribute to viral entry and tropism and therefore represent promising candidates for experimental validation and therapeutic targeting. Our findings highlight the utility of AI-driven pipelines in accelerating the discovery of host factors relevant to pandemic influenza viruses.

## 1. Introduction

The highly pathogenic avian influenza virus (HPAIV) H5N1 is one of the most concerning zoonotic pathogens due to its potential to cause a human pandemic. Since its emergence in 1997, H5N1 has repeatedly spilled over from birds into humans, often causing severe disease, though sustained human-to-human transmission has not yet been established [1,2]. The current wave of H5N1 circulation (2023–2025) has been marked by intense spread among wild and farmed birds, with occasional human cases reported in Chile, the UK, and the USA [3,4,5]. Alarmingly, infections have also been documented in several mammalian species, including minks, seals, and cattle, raising concerns about ongoing adaptation toward human hosts [6,7,8]. The World Health Organization has therefore classified the pandemic risk associated with H5N1 as “high” [9].

A key molecular determinant of influenza host specificity and tropism is the viral hemagglutinin (HA) surface glycoprotein. HA mediates viral entry by binding to sialylated glycans on host cells and subsequently triggering membrane fusion [10]. Mutations in HA that alter receptor-binding specificity or stability have historically underpinned host shifts in avian influenza viruses into humans [11,12]. For example, recent structural analyses demonstrated that specific substitutions in H5N1 HA can shift binding preference from avian-like (α2,3-linked) to human-like (α2,6-linked) sialic acids, potentially facilitating airborne transmissibility [13,14]. Other studies revealed that internal auto-bound glycans within HA must be displaced to permit receptor engagement, adding another layer of regulation to receptor binding [15].

While HA–glycan interactions are essential, they may not fully explain host cell entry. Evidence suggests that influenza viruses can exploit additional host cell surface proteins as attachment factors or co-receptors. For instance, HA has been shown to bind human immune cell subsets in ways not fully dependent on sialylated glycans [16]. Furthermore, multivalent filamentous HA–glycan arrangements can enhance avidity and alter binding geometry, suggesting complex binding modes at the cell surface [17]. However, systematic identification of human cell-surface proteins that directly interact with H5N1 HA remains limited. Although the A/goose/Guangdong/1/1996 strain used here is strictly avian and not expected to infect human airway epithelial cells due to its preference for α2,3-linked sialic acids, the goal of this study is not to model productive infection. Instead, we evaluate the direct structural compatibility between the HA ectodomain and human extracellular proteins. This approach is independent of viral entry competence and allows identification of potential host attachment factors that could be relevant across both avian and human-adapted H5N1 strains. The same workflow can be applied directly to circulating human H5N1 isolates, including clade 2.3.4.4b viruses.

Virus–host protein–protein interactions (PPIs) are crucial for viral replication, immune evasion, and pathogenesis [18]. Although several large-scale studies have mapped PPIs for influenza polymerase and other internal proteins [19], fewer efforts have targeted viral surface glycoproteins such as HA. Identifying host surface proteins that physically interact with HA could yield new insights into viral tropism and identify promising antiviral targets.

Recent advances in artificial intelligence (AI) and structural biology now enable predictive mapping of virus–host interactions at unprecedented resolution. AlphaFold-based pipelines and machine learning models have been successfully applied to predict viral PPIs and host tropism signatures [20,21,22,23]. For influenza, computational approaches including XGBoost, convolutional neural networks, and hybrid feature models have shown promise in predicting human-virus PPIs and classifying host range based on HA sequences [24,25,26]. Yet, most of these approaches have focused on intracellular interactions or broad PPI networks, rather than surface glycoprotein–host protein complexes.

In this study, we developed an AI-driven pipeline integrating structural modeling, docking, and biological filtering to identify human cell-surface proteins with high likelihood of interacting with H5N1 HA. We prioritized candidates based on structural compatibility, cell-surface localization, and relevance to respiratory epithelium. By applying this framework to contemporary H5N1 isolates, we propose a set of novel HA–host interactions that may influence infection dynamics and represent promising targets for antiviral discovery.

## 2. Materials and Methods

Sequence Data Acquisition—The protein sequence of the Influenza A virus (A/goose/Guangdong/1/1996(H5N1)) hemagglutinin (HA) gene was downloaded from NCBI database on 24 January 2024.

All human proteins sequences for cell surface GO annotation number 0009986 (a total of 958 proteins) were downloaded from UniProt database on 14 July 2023 (UniProt Consortium, 2023). Proteins not annotated under this GO term (e.g., intracellular or nuclear proteins such as DCTPP1 or RPSA) were intentionally excluded, as the objective was to identify direct biochemical compatibilities between extracellular prefusion HA and surface-exposed human proteins, not the intracellular interactome.

The AlphaFold 3 databases were downloaded on 1 June 2025.

Bait-Target Pair Generation—Protein sequences from hemagglutinin gene sequences (bait) and every individual human protein from the cell surface GO annotation (targets) were combined into bait-target pairs. For each bait-target pair, an AlphaFold3 JSON file was created.

AlphaFold 3 modeling—AlphaFold 3 [27] version 3.0.1 was run in “data pipeline only” mode for every bait-target JSON file using the parameters “--max_template_date=2025-06-01 --norun_inference”.

AlphaFold 3 was run in “model inference only” for every bait-target JSON file created by AlphaFold 3 data pipeline using the parameters “--norun_data_pipeline”.

The highest ipTM score for each bait-target pair was extracted from each summary confidences JSON file created by AlphaFold 3 model inference and was saved into an Excel file.

AFM-LIS [28] was used to compute Local Interface Score (LIS) and Local Interaction Area (LIA) for each bait-target using the output of AlphaFold 3 as an input for AFM-LIS. The result was saved into an Excel file.

Data Integration and Thresholding—Excel files containing LIS and LIA from AFM-LIS and ipTM scores from AlphaFold 3 were merged. A threshold of 0.203 for LIS and 3432 for LIA were used to color the cells in green to show the best potential interactors. The thresholds are calculated according to Kim et al., 2024 (see Supplementary Figure S8 in Ref. [28]). A positive PPI is suggested if either of the following conditions is met: Best LIS ≥ 0.203 AND Best LIA ≥ 3432, or Average LIS ≥ 0.073 AND Average LIA ≥ 1610.

Image generation—Images were generated using ChimeraX version 1.10, GIMP version 3.0.4 and Inkscape version 1.3.

Functional classification of host interactors—Candidate HA-interacting proteins were manually grouped into six biological categories (immune/inflammatory, adhesion/surface receptors, cytokines/growth factors, neural/synaptic proteins, signaling enzymes, and plasma/structural proteins). Classification was based on UniProt and Gene Ontology functional annotations, domain architecture, tissue expression, and known relevance to viral tropism, immune modulation, and epithelial/endothelial biology.

## 3. Results

### 3.1. Identification of the Top 5 High-Confidence HA–Host Protein Interactions

**Figure 1:**
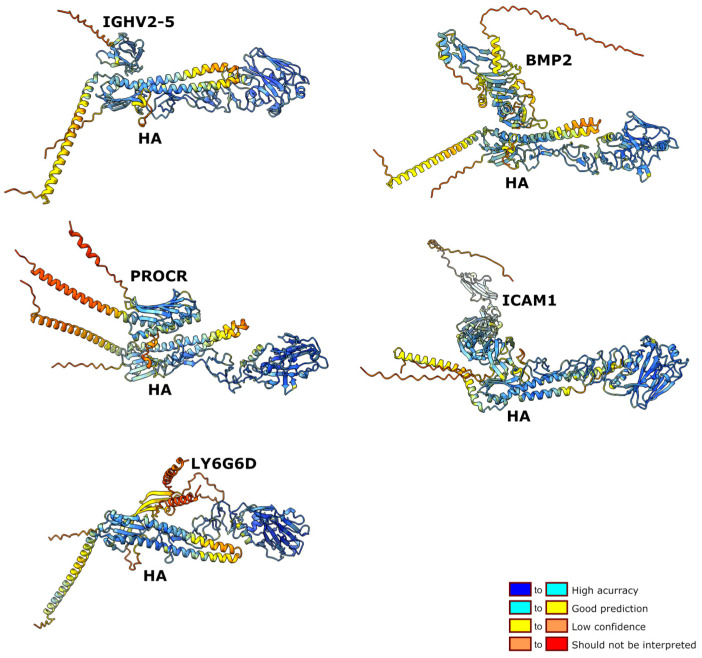
Molecular models of the high-confidence human proteins interacting with HA. The color code indicates the confidence for each domain.

Using AlphaFold 3 (AF3), we screened 987 human extracellular and membrane-associated proteins for potential interactions with the prefusion H5N1 hemagglutinin (HA) protein. AF3 returned 30 high-confidence candidate complexes, among which the top five showed ipTM larger than 0.75 and well-defined interface geometries (Table 1). These top candidates include BMP2, PROCR, ICAM1, LY6G6D, and IGHV2-5. All five are accessible to extracellular HA and play roles in immune regulation, vascular biology, or antigen recognition, processes central to H5N1 pathogenesis. Figure 1 shows structural models of high-confidence human proteins interacting with HA.

### 3.2. The H5N1 HA-Interacting Proteins Form Six Functional Groups

The H5N1 HA-interacting proteins can be regrouped in six functional groups shown in Table 1, namely Immune/Inflammatory, Surface/Adhesion, Growth Factors, Neural/Synaptic, Signaling Enzymes, and Plasma/Structural. These groups reflect major processes disrupted during severe influenza and H5N1 disease Details for each group are provided in Table 1. Of note, the six categories represent biological interpretation and are not derived from a clustering algorithm.

### 3.3. Structural and Functional Annotation of the Remaining 26 Predicted Interactors

The remaining 25 predicted HA-binding proteins were annotated for function, disease links, expression, structural features, and, importantly, subcellular localization (Table 2). Most proteins are located at the plasma membrane or secreted into the extracellular space, consistent with accessibility to intact HA during viral attachment and early entry.

### 3.4. Subset of Predicted Interactors Overlaps with Known Influenza Host Factors

Although few human proteins have been experimentally shown to bind HA directly (e.g., NKp44, NKp46), several AF3-predicted partners have strong independent evidence linking them to influenza A infection, host restriction, or influenza-induced lung injury. These proteins -BTN3A3, ICAM1, CEACAM19 (via CEACAM1), CSF3R, LBP, and HRG- form a coherent subset of high biological plausibility (Table 3). Their presence within the predicted interactome reinforces that AF3 captures relevant immunological and pathophysiological themes, not random structural matches.

### 3.5. Localization Strengthens Biological Plausibility

We systematically added subcellular localization for all host interactors to refine biological plausibility. The majority of predicted interactors are plasma membrane or secreted proteins, and therefore physically accessible to intact prefusion HA during the initial attachment phase. This is consistent with the goal of modeling extracellular HA binding rather than intracellular viral processes. Predicted intracellular interactors are interpreted cautiously, as possible downstream signaling interfaces, post-entry partners, or structurally compatible false positives.

This information is now integrated throughout Table 1, Table 2 and Table 3.

These classes are not random; instead, they map onto known influenza mechanisms including epithelial damage, vascular leakage, neutrophil-driven pathology, cytokine amplification, and coagulopathy.

## 4. Discussion

The SARS-CoV-2 (COVID 19) pandemic revealed how unprepared our medical systems were to face large scale viral outbreaks, in this particular case leading to dramatic issues at various levels of our societies. Strong of this lesson, we now understand that it is essential to be prepared for the next such pandemics, in case it occurs. The H5N1 virus has been identified as a putative threat to humanity because it already replicates in animal and human cells. We are possibly at only a few mutations away of a highly dangerous infection spreading across the world.

One valuable method to prepare for H5N1 spread is to identify and characterize surface proteins in human cells that can be targeted by the virus, namely its surface antigens such as HA. In this study, we have used the software package AlphaFold 3, a performant software for determining the structures of proteins as well as protein–protein interactions. All human surface proteins were virtually paired with H5N1 HA and interactions found positive were selected (see Section 2). In our conditions, a total of 30 human proteins were found positive in this assay. Five of them have interaction scores that overpass the 25 others. We were able to manually separate interaction pairs into six categories (see Table 1). A detailed analysis of the selected proteins emphasizes the functional variety of HA interactors. Overall, these interactors are specific proteins in distinct cell types, and they have many specific structural domains (also see Figure 1). Moreover, their role in H5N1 infection was in many cases predictable. This last point is important as pharmacologists and clinicians can find in this work a wealth of information useful to develop drugs and treatments for facing a possible H5N1 outbreak in humans. Similar studies involving PPI maps have revealed drug candidates for SARS-CoV-2 [58].

AF3 reveals a structured, biologically coherent HA–host interactome. Although AF3 predictions do not establish physical binding, several aspects of our results indicate that the predicted interactome is biologically meaningful rather than random. High ipTM scores for the top-ranked complexes suggest well-formed HA–host interfaces. The proteins fall into clear functional groups consistent with processes known to be critical in H5N1 pathogenesis: immune sensing, epithelial barrier control, vascular integrity, neutrophil biology, and coagulation. This alignment argues that AF3 is capturing real structural compatibilities relevant to influenza infection.

Avian HA and human-cell infectivity: scope of the structural predictions. Because the HA used in this study derives from an avian (goose) H5N1 isolate, it is not expected to efficiently enter human epithelial cells due to its specificity for α2,3-linked sialic acids. However, the aim of our computational screening was not to model productive infection, but to evaluate direct structural compatibility between extracellular prefusion HA and human surface proteins. Protein–protein docking scores are not dependent on viral entry efficiency, and the prefusion HA fold is highly conserved across avian and emerging human-lineage H5N1 viruses. Our predictions therefore reflect potential biochemical compatibilities that may become relevant if human-adapting mutations arise. We also note that the same workflow can be applied without modification to human H5N1 isolates as sequence data become available.

Independent host-factor evidence reinforces several predictions. Six AF3-predicted proteins already have independent experimental links to influenza A infection (Table 3). BTN3A3 restricts avian IAV replication in primate airways; ICAM1 and CEACAM1 are induced in influenza-infected cells; CSF3R signaling exacerbates influenza-associated lung injury; LBP mediates lethal TLR4-dependent inflammation; HRG regulates vascular and coagulation pathways disrupted in severe flu. The presence of this non-random overlap strengthens the credibility of AF3-generated predictions.

Complementarity between AI predictions and cellular proteomics. A recent proteomic study [59] identified DCTPP1 and RPSA as HA-associated host proteins. These were not present in our predicted set because they were not included in the initial protein pool, which was restricted to the Gene Ontology category “cell surface proteins.” DCTPP1 and RPSA are intracellular proteins and therefore fall outside the biological scope of our extracellular HA binding analysis. Their detection in proteomics highlights interacting partners that may be relevant after viral internalization or as part of intracellular multi-protein complexes. Thus, proteomic and structural AI-based predictions capture distinct but complementary layers of the HA interactome.

HA processing and antigen presentation: scope and limitations. HA is not intact throughout infection. HA0 is cleaved into HA1 and HA2, undergoes pH-triggered rearrangements during endocytosis, and is ultimately digested into peptides for MHC I/II presentation. Our AF3 analysis intentionally focuses on extracellular prefusion HA, which is the relevant conformation for virus attachment and entry. We explicitly do not model post-fusion HA, HA degradation, MHC loading, or T-cell recognition. This limitation is acknowledged.

In addition, several predicted interactors, such as immunoglobulin variable segments, CEACAM family members, and plasma proteins including HRG, are expected to bind intact HA ectodomain rather than peptide fragments derived from antigen processing. Our analysis therefore focuses on the extracellular, prefusion form of HA relevant for receptor engagement, immune attachment factors, or soluble scavenging proteins. Epitope-level screening of HA peptides represents a valuable future extension of our computational framework.

Localization considerations clarify which interactions are most plausible. We added a Localization column to Table 2 and Table 3. Most high-confidence hits are extracellular or plasma-membrane proteins, i.e., physically accessible to HA. These interactions are the most plausible. Intracellular predictions are interpreted as lower-confidence hypotheses or possible downstream effectors. Localization thus adds an important layer of biological filtering.

Constraints on experimental validation (BSL-3 requirements). Validating and confirming 30 HA–host interactions involving highly pathogenic H5N1 is not feasible without BSL-3 containment and a dedicated virology program. Our study serves as a computational roadmap, identifying priority targets for future biochemical and virological validation rather than delivering validated interactions. Given the above constraints, our AF3 structural predictions provide: (i) High-confidence candidate complexes that can guide targeted experimental programs, (ii) Functional clustering consistent with known influenza biology, (iii) A subset of host factors already validated in independent influenza studies, and (iv) A structurally guided hypothesis framework for viral entry, immune evasion, and pathogenesis. We present these findings as a preliminary but biologically grounded interactome map, offering new testable hypotheses for future influenza research.

## 5. Conclusions

The computational workflow presented here (integrating sequence retrieval, structural prediction, and systematic screening of human surface proteins) offers a rapid approach to identify putative extracellular interactors of viral glycoproteins. While our results do not establish physical binding and must be validated experimentally, they provide a structured and biologically coherent set of hypotheses for future influenza research. Importantly, the workflow is, in principle, applicable to other viral surface proteins, although such extensions remain to be demonstrated experimentally. Our findings thus serve as a foundation for targeted virological studies aimed at clarifying mechanisms of viral attachment, tropism, and pathogenesis in emerging influenza strains.

## Figures and Tables

**Table 1:** Functional grouping of predicted HA–host interactors.

Group	Representative Proteins	Functional Axis	H5N1 Connection
Immune/Inflammatory	ICAM1, IL12RB1, IL15, SLAMF1, KLRB1	Cytokine and immune signaling	Cytokine storm, immune modulation
Surface/Adhesion	PROCR, CEACAM19, LY6G6D	Viral entry and cell binding	Potential alternative HA interactions
Growth Factors	BMP2, AIMP1, EPO	Tissue repair, host growth regulation	Host remodeling post infection
Neural/Synaptic	LRFN2, GPR37	Neurotropic or cross-reactive pathways	Potential neurological sequelae
Signaling Enzymes	RPS6KB1, PTPRT	Intracellular signaling regulation	Virus-induced signaling reprogramming
Plasma/Structural	FGB, HRG, CSF3R	Coagulation, barrier integrity	Vascular leakage, sepsis-like features

**Table 2:** Remaining 26 significant HA interactors.

Symbol	Function/Role	Disease Links	Expression	Localization	Structural Notes	Key Reference
IGHV1-3	Immunoglobulin heavy variable segment shaping antigen specificity.	Autoimmunity, infection, B-cell malignancy.	B cells/plasma cells	Plasma membrane/secreted	Ig V-domain; CDR loops define binding.	[29]
IGHV3-30-3	Ig heavy variable segment (antigen binding).	As above.	B-cell lineage	Plasma membrane/secreted	Ig V-domain	[29]
EPO	Cytokine driving erythropoiesis via EPOR.	Anemia, polycythemia, CKD; tumors.	Kidney interstitial cells; secreted to plasma	Secreted	4-helix bundle cytokine	[30]
IGHV3-30	Ig heavy variable segment.	As above.	B-cell lineage	Plasma membrane/secreted	Ig V-domain	[29]
IGHV3-30-5	Ig heavy variable segment.	As above.	B-cell lineage	Plasma membrane/secreted	Ig V-domain	[29]
IL12RB1	IL-12/23 receptor β1 subunit; Th1/NK signaling.	MSMD immunodeficiency.	T cells, NK cells, DCs	Plasma membrane (type I receptor)	FN3 domains	[31]
IL15	NK and CD8 T-cell homeostasis; transpresentation by IL15RA.	Autoimmunity, inflammation, cancer.	APCs, stromal and epithelial cells	Secreted	4-helix bundle cytokine	[32]
PTPRT	Receptor-type tyrosine phosphatase; tumor suppressor.	CRC, brain tumors.	Neuronal and epithelial	Plasma membrane (Ig/FN3 ectodomain)	Dual PTP domains	[33]
BTN3A3	Butyrophilin family immune modulator.	Viral and tumor immunity.	APCs, epithelia	Plasma membrane (Ig-like ectodomain)	B30.2/PRY-SPRY domain	[34]
LRFN2	Synaptic adhesion; excitatory synapse assembly.	Neurodevelopmental disorders.	Neurons	Plasma membrane (LRR/FN3)	TM, short tail	[35]
HRG	Histidine-rich glycoprotein; coagulation/immunity.	Sepsis, thrombosis, cancer.	Liver plasma protein	Secreted	Cystatin-like domains; Zn^2+^	[36]
TNFRSF11A (RANK)	RANK receptor; bone and immune signaling.	Osteopetrosis, Paget’s disease, metastasis.	Osteoclast precursors, DCs, epithelia	Plasma membrane (TNFR)	Cysteine-rich domains	[37]
RPS6KB1	p70S6K; mTORC1 effector.	Cancer (oncogenic activation).	Broad, high in proliferating cells	Cytosolic	Ser/Thr kinase (Thr389)	[38]
CRLF2	TSLP receptor subunit; B-cell development.	B-ALL; allergy.	Hematopoietic progenitors, thymic stroma	Plasma membrane	Type I cytokine receptor	[39]
CEACAM19	CEACAM family adhesion molecule.	Epithelial cancers.	Epithelium (predicted)	Plasma membrane	Ig-like ectodomains	[40]
LRFN5	Synaptic adhesion; neuronal connectivity.	Neurodevelopmental traits.	Neurons	Plasma membrane (LRR)	TM	[41]
TMC1	Mechanotransduction channel component.	DFNB7/11 deafness.	Cochlear hair cells	Multi-pass membrane	Channel-like protein	[42]
GPR37	GPCR in neuronal/myelin biology; parkin substrate.	Parkinson’s, leukodystrophy.	Oligodendrocytes, neurons	Plasma membrane (GPCR)	Class A, 7TM	[43]
CD200R1L	CD200 receptor-like immune modulator.	Immune regulation.	Myeloid lineage (pred.)	Plasma membrane	Ig-like ectodomains; TM	[44]
KLRB1	C-type lectin-like NK/T-cell receptor.	Autoimmunity, infections, cancer.	NK, NKT, CD4/CD8 T-cells	Plasma membrane (type II)	CTLR homodimer	[45]
LBP	LPS-binding protein for TLR4 signaling.	Sepsis, infection susceptibility.	Liver, plasma; GI epithelia	Secreted	Lipid-binding protein	[46]
CSF3R	G-CSF receptor; granulopoiesis.	CNL/AML; neutropenia.	Myeloid progenitors, neutrophils	Plasma membrane (type I receptor)	Cytokine receptor	[47]
HHIP	Hedgehog-interacting protein.	COPD, cancer, development.	Lung, cartilage	Secreted	Hh-binding protein	[48]
SLAMF1	SLAM-family lymphocyte activator.	Measles entry receptor; immune regulation.	Activated T/B cells, DCs, macrophages	Plasma membrane (IgV/IgC)	ITSM motifs	[49]
FGB	Fibrinogen β-chain.	Dysfibrinogenemia, thrombosis, CVD.	Liver → plasma	Secreted	Fibrinogen domains	[50]
AIMP1	tRNA synthetase scaffold; secreted stress cytokine-like factor.	Inflammation, angiogenesis, cancer.	Ubiquitous; stress secretion	Secreted/cytosolic	Adaptor; extracellular processed	[51]

**Table 3:** Host factors with independent influenza-related evidence.

Protein	Localization	Influenza-Related Evidence	Key Reference
BTN3A3	Plasma membrane	Primate-specific antiviral restriction factor inhibiting avian IAV replication; expressed in human airway cells.	[52]
ICAM1	Plasma membrane	Upregulated in influenza-infected airway epithelial cells; modulates viral survival and cytokine responses.	[53]
CEACAM19 (via CEACAM1)	Plasma membrane	CEACAM1 highly induced in H5N1-infected alveolar type II cells; enhances inflammatory signaling.	[54]
CSF3R	Plasma membrane	CSF3–CSF3R axis drives neutrophilic lung injury and barrier disruption in influenza.	[55]
LBP	Secreted/plasma	TLR4–LBP pathway mediates lethal influenza-induced acute lung injury; Eritoran protection in vivo.	[56]
HRG	Secreted/plasma	Regulates coagulation, vascular integrity, and immune responses—pathways disrupted in severe influenza.	[57]

## Data Availability

The original contributions presented in this study are included in the article. Further inquiries can be directed to the corresponding author.

## Abbreviations

The following abbreviations are used in this manuscript:

AF3	AlphaFold 3
AI	Artificial Intelligence
GO	Gene Ontology
HA	Hemagglutinin Protein
Ig	Immunoglobulin
IL	Interleukin
LIA	Local Interface Area
LIS	Local Interaction Score
MHC	Major Histocompatibility Complex
PPI	Protein–Protein Interaction
